# From gut microbiota-driven mucosal barrier disruption to myeloid-dominant immunosuppressive niches in colorectal cancer

**DOI:** 10.3389/fimmu.2026.1932590

**Published:** 2026-08-21

**Authors:** Na Lu, Lei Zhang, Hanyu Sun, Yuwenshu Zhou, Panli He, Changrui Zhao, Yue Wang

**Affiliations:** 1 Research Center of Traditional Chinese Medicine and Clinical Pharmacy, Shandong Provincial Maternal and Child Health Care Hospital Affiliated to Qingdao University, Jinan, China; 2 School of Public Health, Shenyang Medical College, Shenyang, China; 3 The First Clinical Medical College, Shenyang Medical College, Shenyang, China

**Keywords:** colorectal cancer, gut microbiota, immunotherapy resistance, microsatellite stable colorectal cancer, mucosal barrier, myeloid-derived suppressor cells, tumor-associated macrophages

## Abstract

Colorectal cancer (CRC) develops within a mucosal ecosystem in which epithelial barrier function, colonizing microbes, inflammatory signaling, and immune cell composition are closely interdependent. Immune checkpoint inhibitors have significantly altered treatment outcomes in patients with mismatch repair-deficient or microsatellite instability-high (dMMR/MSI-H) CRC, but most microsatellite-stable (MSS) tumors still respond poorly, and this is not solely due to lower antigenicity. This article explains this gap mechanistically: gut microbiota dysbiosis weakens the mucosal barrier, promotes microbial translocation, amplifies cytokine and eicosanoid signaling, and remodels the tumor microenvironment into an immunosuppressive state dominated by myeloid cells. We focus on *Fusobacterium nucleatum* and enterotoxigenic *Bacteroides fragilis*, microbial metabolites, tight junction disruption, and the IL-6/STAT3, NF-κB, TNF-alpha, and COX-2/PGE2 pathways that link epithelial stress with innate immune remodeling. Downstream processes include tumor-associated macrophages, myeloid-derived suppressor cells, neutrophil polarization, dendritic cell dysfunction, regulatory T cells, cancer-associated fibroblasts, CXCL12-mediated T-cell exclusion, and hypoxia. These processes collectively create a spatially structured drug-resistance niche, preventing effector T cells from functioning effectively, rather than simply resulting in an immunologically “cold” tumor. We believe that integrating this microbiota-barrier-myeloid axis into biomarker development and mechanism-matched combination therapy design offers a more rational and promising approach to overcoming immunotherapy resistance in CRC, especially in MSS disease, compared to empirical drug combinations.

## 1 Introduction

Colorectal cancer (CRC) remains one of the most common malignancies worldwide and a major cause of cancer-related death (1). Its clinical behavior cannot be explained solely by epithelial mutations or anatomical staging. CRC occurs on long-exposed mucosal surfaces where diet, microbiome, epithelial barrier integrity, inflammatory status, matrix remodeling, and immune surveillance continuously interact (2). This ecological perspective is particularly important in the era of immunotherapy. Antibodies targeting PD-1 have generated durable responses in mismatch repair-deficient or microsatellite instability-high (dMMR/MSI-H) CRC, making checkpoint blockade the standard treatment option for this molecular subgroup (3, 4). In contrast, most microsatellite-stable (MSS) CRCs, even with seemingly targetable immune and inflammatory features, rarely benefit from single-agent checkpoint inhibition (5). A useful way to understand this gap is to differentiate between immune visibility and immune permission. Consensus molecular subtypes, matrix enrichment, inflammatory signals, and immune cell organization patterns collectively determine whether antigen-specific T cells can enter, survive, and function in CRC tissues (6). In many tumors, immune tolerance is not merely a lack of antigenicity, but reflects a spatially organized inhibitory niche that blocks effective T-cell immunity. This article focuses on an interconnected mechanism: gut microbiota dysbiosis driving mucosal barrier disruption, microbial translocation, amplification of chronic inflammation, and myeloid-dominated immune remodeling. Our goal is to integrate these processes into a CRC-specific immune escape model, with a focus on MSS, a disease with the greatest therapeutic need.

## 2 Gut microbiota dysbiosis in colorectal cancer

Metagenomic studies have repeatedly identified CRC-related microbial signatures, although the specific microbial taxa and effect sizes vary depending on the cohort, region, diet, sampling method, and tumor location. Two large-scale fecal metagenomic meta-analyses conducted by Wirbel et al. and Thomas et al. in 2019 independently converged on reproducible CRC-associated signatures across cohorts and identified a link with choline degradation (7, 8). These signatures should not be interpreted as evidence of a single causal dysbiotic state that replaces a healthy microbiome. A more plausible explanation is that CRC-related microbial dysbiosis represents a functional shift, a change in the combination of microbes and metabolites that favors epithelial stress, genotoxicity, altered nutritional processing, and inflammatory states (9).

*Fusobacterium nucleatum* is the most well-studied example. In 2012, Castellarin et al. and Kostic et al. published two independent papers in Genome Research, first demonstrating that *F. nucleatum* was enriched in human CRC tissues (10, 11). Subsequently, Kostic et al. further demonstrated that *F. nucleatum* could enhance intestinal tumorigenesis and remodel the tumor immune microenvironment in *Apc^Min/+^* mice (12). The relevant mechanisms are now quite specific. FadA binding to E-cadherin activates beta-catenin signaling in epithelial cells, while Fap2 binding to the inhibitory receptor TIGIT weakens natural killer- and T-cell-mediated cytotoxicity, thereby promoting tumor cell proliferation and immune evasion (13, 14).

Evidence concerning *F. nucleatum* also suggests that dysbiosis should be understood dynamically rather than as a static biomarker. Yu et al. linked it to chemotherapy resistance mediated by autophagy regulation; Bullman et al. showed that live *Fusobacterium* can persist in patient-derived liver metastases and that antibiotic treatment can reduce xenograft tumor growth, suggesting that tumor-associated bacteria can disseminate with malignant ecosystems rather than merely colonizing tumors at a late stage (15, 16). Recent studies have further refined this picture: Zepeda-Rivera et al. found that CRC is enriched in a specific *F. nucleatum animalis* clade, rather than the entire species (17); Jiang et al. linked succinic acid from *F. nucleatum* to immunotherapy resistance caused by weakened cGAS-STING signaling (18). However, not all interactions go in the same direction. Wang et al. reported that under certain conditions, *F. nucleatum* can promote the response of MSS CRC to PD-1 therapy, reminding us that strain, anatomical compartment, metabolic state, and host background can all change the direction of the effect (19). These findings indicate that *F. nucleatum* should not be regarded as uniformly tumor-promoting; its effects are strain- and context-dependent. Other microorganisms further extend this axis. Enterotoxigenic *Bacteroides fragilis* (ETBF) can promote colonic tumorigenesis through Th17-related inflammatory programs, as first demonstrated by Wu et al. (20). Dejea et al. described an invasive multimicrobial biofilm in familial adenomatous polyposis, supporting the idea that spatially organized communities, rather than isolated species, influence mucosal damage (21). Chung et al. elucidated how *B. fragilis* toxin triggers a pro-cancer inflammatory cascade in the colonic epithelium, while Cao et al. linked ETBF to malignant transformation mediated by altered exosomal miR-149-3p signaling (22, 23). Microbial metabolites can have divergent effects. As O’Keefe et al. showed in a comparison between African American and rural African populations, short-chain fatty acids derived from dietary fiber generally support epithelial homeostasis, whereas Yang et al. demonstrated that high-fat-diet-induced changes in the microbiota and bile acid profiles promote tumorigenesis (24, 25). Thus, microbiota-derived effects can be protective or harmful depending on dietary and metabolic context.

## 3 Mucosal barrier disruption and microbial translocation

The intestinal barrier is not a passive wall. As recently reviewed by Neurath, Artis, and Becker, it is an active immunological interface comprised of mucus, epithelial cells, tight junctions, antimicrobial peptides, secretory IgA, matrix cues, and resident immune cells (26). In CRC, barrier dysfunction can occur before or during tumorigenesis and is subsequently amplified by tumor-derived inflammation. Loss or redistribution of tight junction proteins such as ZO-1, claudins, and occludin increases permeability, allowing intraluminal products to reach epithelial, matrix, and immune compartments that are normally isolated from high microbial loads. Thus, the mucosa is exposed to lipopolysaccharides, flagellins, bacterial DNA, extracellular vesicles, and damage-associated molecular patterns from stressed or dying cells. Barrier disruption is important because it translates dysbiosis into immune remodeling. Translocated microbial products activate pattern recognition receptors on epithelial cells, dendritic cells, macrophages, neutrophils, endothelial cells, and fibroblasts. The result is not necessarily effective antitumor immunity. Sustained low-level sensing tends to promote stress-induced myelopoiesis, tolerogenic antigen presentation, epithelial survival signals, and the recruitment of suppressive innate immune cells. In early lesions, these signals may support tissue-repair programs that limit acute injury; in established CRC, the same programs protect malignant epithelium, drive angiogenesis, and isolate cytotoxic lymphocytes. In other words, the timing of barrier failure may determine whether microbial sensing protects the host or the tumor.

The barrier also links histology and molecular typing. Tumor ulceration, mucus depletion, biofilm localization, weakened tight junction staining, and bacterial products in the tumor or peritumoral matrix all indicate that microbial signals have escaped the intestinal lumen. These features are not interchangeable. Tumors rich in invasive bacteria may require a different treatment logic than those dominated by soluble inflammatory mediators or ischemic hypoxia. Therefore, future CRC research should pair fecal or tissue microbiome analysis with epithelial barrier markers, spatial immunophenotypes, and blood-based translocation evidence, rather than treating dysbiosis as a single compartmental measure.

**Figure 1:**
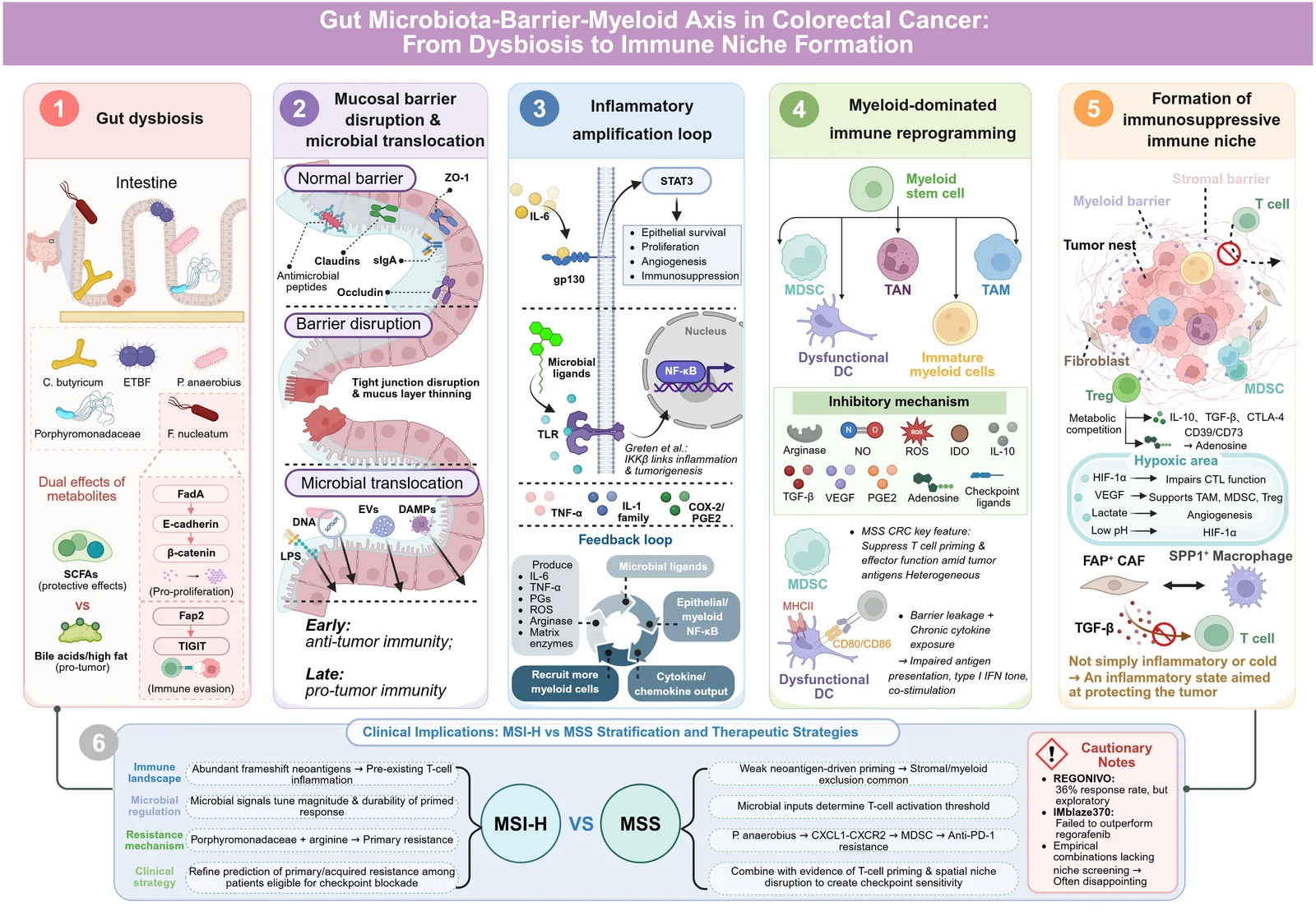
Gut microbiota-barrier-myeloid axis in colorectal cancer immunosuppression.

This distinction is crucial for the microbiota-barrier-myeloid axis. Acute microbial sensing may initiate antitumor immunity, while persistent microbial translocation can promote both adaptive and inhibitory responses: epithelial cells become less responsive to danger signals, dendritic cells lose their co-stimulatory capacity, and myeloid cells acquire tissue repair and immunomodulatory phenotypes. In microsatellite-stable CRC, neoantigen-driven T-cell pressure is typically weaker than in MSI-H disease, thus barrier-related innate inflammation may shift the balance from tumor rejection to immune suppression (**Figure 1**).

Importantly, microbiota-dependent modulation is not confined to MSS CRC. In MSI-H/dMMR tumors, abundant frameshift neoantigens and pre-existing T-cell inflammation usually provide a substrate for checkpoint blockade, so microbial signals predominantly tune the magnitude and durability of an already primed response. A longitudinal multi-omics study of 77 patients with MSI-H/dMMR gastrointestinal cancers, validated in 39 patients, linked Porphyromonadaceae and circulating arginine to primary resistance, showing that favorable tumor genetics do not eliminate microbiome-mediated resistance (27). Consistently, *Peptostreptococcus anaerobius* induced resistance to anti-PD-1 therapy in the immunotherapy-responsive MC38 allograft model through CXCL1-CXCR2-dependent MDSC recruitment (28). Conversely, *Fusobacterium nucleatum* has been reported to increase STING/NF-κB signaling, PD-L1 expression, and IFN-γ+ CD8+ T-cell accumulation during PD-1/PD-L1 blockade (29), whereas *F. nucleatum*-derived succinate can suppress cGAS-STING signaling and promote resistance (18). These apparently opposing observations support a strain-, metabolite-, compartment-, and host-dependent model rather than a uniform species effect. In MSS CRC, where neoantigen-driven priming is usually weaker and stromal/myeloid exclusion more common, the same microbial inputs are less likely to generate checkpoint-responsive immunity and more likely to determine whether the tumor remains below or crosses the threshold for productive T-cell activation. Thus, the difference between MSI-H and MSS CRC is best viewed as a difference in baseline immune context and effect size, not as the presence versus absence of microbiome influence. However, direct head-to-head studies comparing the effects of identical microbial taxa or metabolites on immune checkpoint inhibitor responses in MSI-H versus MSS CRC remain scarce; this comparison should therefore be regarded as a mechanistic synthesis rather than an established subtype-specific causal hierarchy.

## 4 Inflammatory amplification loops

Once the barrier weakens, inflammatory signals can become amplified. IL-6/STAT3 is a key node because it connects microbial and cytokine sensing with epithelial survival, proliferation, angiogenesis, and immunosuppression. Grivennikov et al. demonstrated in a colitis-associated cancer model that IL-6 and STAT3 maintain the survival of precancerous epithelial cells, showing how repair signals can be transformed into pro-tumorigenic signals (30). NF-κB provides a second bridge between innate immune activation and carcinogenesis. Greten et al.’s classic study showing that IKKβ links inflammation and tumorigenesis remains important because it distinguishes the contributions of epithelial and myeloid cells to tumor initiation and growth (31). TNF-alpha, IL-1 family cytokines, chemokines, and COX-2/PGE2 further stabilize this circuit. Wang and DuBois elucidated how eicosanoid signaling promotes proliferation, angiogenesis, immunosuppression, and resistance to apoptosis in CRC (32). These pathways do not operate in isolation. Microbial ligands induce epithelial and myeloid NF-κB activity; NF-κB and STAT3 increase cytokine and chemokine output; cytokines recruit more myeloid cells; these myeloid cells then produce more IL-6, TNF-alpha, prostaglandins, reactive oxygen species, arginase, and matrix-remodeling enzymes. The feedback loop is both biochemical and spatial, concentrated at the invasion front, ulcerated mucosa, hypoxic areas, and microbial biofilms.

Recent microbiome-immunotherapy studies have further reinforced this model, and more importantly, they have shown that microbial effects can be bidirectional. Liu et al. found that *Peptostreptococcus anaerobius* drives anti-PD-1 resistance and exacerbates CRC through myeloid-derived suppressor cells in mice (28). Conversely, Xie et al. showed that tumor-resident *Clostridium butyricum* can enhance anti-PD-1 efficacy by inhibiting IL-6-mediated immunosuppression (33). Van Baarle et al. added an unexpected axis, showing that IL-1R-driven enteric glia-macrophage interactions enhance pro-tumor inflammation (34). Overall, these studies suggest that inflammatory amplification is not background noise in CRC; it can determine whether microbial signals ultimately translate into immune rejection or immune tolerance.

## 5 Myeloid-dominant immune reprogramming

Myeloid cells are the primary interpreters of the disrupted mucosal ecosystem. Zhang et al. used single-cell analysis of colon cancer to show that therapy targeting myeloid cells can remodel the state of tumor macrophages and monocytes, demonstrating that these populations are plastic rather than fixed (35). In CRC, tumor-associated macrophages (TAMs), monocytic and polymorphonuclear MDSCs, tumor-associated neutrophils, immature myeloid cells, and dysfunctional dendritic cells synergistically suppress adaptive immunity through the expression of arginase, nitric oxide, reactive oxygen species, indoleamine 2,3-dioxygenase, IL-10, TGF-beta, VEGF, prostaglandins, adenosine, and checkpoint ligands.

MDSCs are particularly important for MSS CRC because they can suppress T-cell priming and effector function even in the presence of tumor antigens. As Veglia, Sanseviero, and Gabrilovich have highlighted, MDSCs are heterogeneous and overlap with inflammatory monocyte and neutrophil-like states at the transcriptional level, thus functional experiments and spatial context are crucial before labeling a cell as suppressive (36). Neutrophils are also plastic. Jaillon et al. reviewed evidence showing that pro-tumor neutrophilic programs can support angiogenesis, matrix remodeling, metastasis, and T-cell suppression, while other states contribute to acute tumor control (37). The phrase “myeloid-dominated niche” should not imply a single cell type; it describes an ecosystem in which innate immune cells outnumber or functionally suppress effective antitumor lymphocyte responses. Dendritic cell dysfunction is another often-underestimated link. Barrier leakage and chronic cytokine exposure can impair effective antigen presentation, type I interferon tone, and co-stimulation. Dendritic cells may fail to effectively prime cytotoxic T cells, adopt tolerogenic states, or become spatially separated from tumor antigens and T cells. This can create a bottleneck upstream of checkpoint blockade. If T cells are under-activated, excluded, subjected to metabolic stress, or surrounded by suppressive myeloid cells, PD-1/PD-L1 blockade lacks a suitable substrate. Therefore, myeloid reprogramming provides a concrete explanation for why microbial and inflammatory CRC features are often associated with poor responses to immunotherapy.

## 6 Suppressive immune niche formation

In colorectal cancers (CRCs), inhibitory niches are not only compositionally determined but also spatially organized. Two landmark spatial studies from 2020 to 2021 exemplified this: Pelka et al. mapped the spatially organized multicellular immune hubs in human CRCs, and Schurch et al. showed that coordinated cellular neighborhoods at the invasion front are associated with antitumor or inhibitory programs (38, 39). In favorable conditions, dendritic cells, activated T cells, and antigen-presenting macrophages form immune hubs; in resistant conditions, T cells are excluded from epithelial nests or trapped behind the stromal and myeloid barriers. This is precisely why overall immune markers can be misleading: two tumors with similar CD8-positive cell densities can differ drastically in whether these cells actually contact the malignant glands, receive co-stimulation, or encounter inhibitory macrophages and fibroblasts.

Cancer-associated fibroblasts (CAFs) are key organizers of T-cell exclusion. Qi et al. used single-cell and spatial analysis to reveal a reinforcing interaction between FAP-positive fibroblasts and SPP1-positive macrophages in CRC, suggesting that the matrix and myeloid programs can form a stable immunosuppressive structure (40). A matrix rich in TGF-beta is particularly important. Tauriello et al. showed that TGF-beta drives immune escape in a genetically reconstituted CRC metastasis model; Calon et al. previously demonstrated that a matrix TGF-beta program supports metastasis initiation (41, 42). In other words, microbes and inflammatory cues do not stop at immune cells; they also activate fibroblast and matrix remodeling programs, thereby physically restricting T-cell entry.

Spatial transcriptomic data strengthen this architectural interpretation. Qi et al. integrated Visium spatial transcriptomics with single-cell RNA sequencing and demonstrated co-localization of FAP-positive fibroblasts and SPP1-positive macrophages in fibrotic tumor regions, with relative depletion of cytotoxic lymphocytes; this program was linked to predicted chemerin, TGF-beta, and IL-1 signaling. High FAP or SPP1 expression was additionally associated with poorer anti-PD-L1 outcomes in the external IMvigor210 bladder cancer cohort, providing cross-tumor rather than CRC-specific supportive evidence (40). Valdeolivas et al. analyzed 14 sections from seven patients and showed that CRC contains intratumoral mixtures of consensus molecular subtype programs rather than spatially uniform subtypes. In CMS2-dominant tumors, immune cells were concentrated mainly in the stroma, while SPP1-positive macrophages and myofibroblasts were enriched in fibrotic regions; spatial ligand-receptor analysis also implicated PLAU-PLAUR communication between myofibroblasts and macrophages or conventional dendritic cells at tumor-stroma interfaces (43). Together with the immune hubs and cellular neighborhoods identified by Pelka et al. and Schurch et al. (38, 39), these data support a model in which suppressive myeloid and fibroblast states are arranged as physical and signaling barriers around malignant glands. However, direct co-mapping of microbial taxa, barrier injury, and host transcripts in the same CRC section remains sparse, and spatial evidence for transcriptionally defined MDSC and neutrophil states is less mature than that for TAM-fibroblast niches. The microbiota-barrier-myeloid axis should therefore be presented as a testable spatial model rather than a fully established causal sequence.

Hypoxia adds another layer of complexity. Abnormal angiogenesis and rapid growth stabilize HIF-1alpha, increase VEGF, drive lactate accumulation, and decrease extracellular pH. These changes impair cytotoxic T-cell function while supporting TAMs, MDSCs, Tregs, angiogenesis, and matrix remodeling. Tregs further suppress immunity through IL-10, TGF-beta, CTLA-4, CD39/CD73-mediated adenosine, and metabolic competition. The resulting niche is not simply inflammatory or cold; it is an inflammatory state aimed at protecting the tumor. This distinction is crucial for MSS CRC. The problem may not lie in increasing nonspecific immune stimulation, but in dismantling spatial barriers, metabolic restrictions, and myeloid-stromal loops that prevent existing or induced T cells from functioning effectively.

## 7 Clinical implications and immunotherapy resistance

The comparison between MSI-H and MSS CRC illustrates why mechanism-matched combination therapy is needed. MSI-H CRC is more likely to respond to PD-1 blockade due to mismatch repair defects generating abundant neoantigens and is often accompanied by an inflammatory T-cell microenvironment (3, 4). MSS CRC typically combines lower immunogenicity with stromal exclusion, suppressive myeloid infiltration, altered antigen presentation, angiogenesis and hypoxic stress, and microbiota-associated inflammation (5). The activity of nivolumab in dMMR/MSI-H CRC in CheckMate 142 and the superiority of pembrolizumab in MSI-H metastatic disease in KEYNOTE-177 demonstrate the value of checkpoint inhibition in the right immune context (3, 44). Results from broader combination-therapy trials have been less encouraging. REGONIVO reported an objective response rate of 36% with regorafenib in combination with nivolumab in a small CRC cohort, but this study is exploratory and should be interpreted with caution (45). Subsequently, IMblaze370 showed that atezolizumab combined with cobimetinib failed to outperform regorafenib in previously treated metastatic CRC (46). The message is clear: empirical combination therapies lacking niche screening are often disappointing, and even small, positive single-arm trials in favorable populations can be seriously misleading.

The microbiota-barrier-myeloid framework suggests specific directions for therapeutic conversion strategies. First, biomarkers should integrate multiple compartments rather than measuring a single indicator in isolation. A composite biomarker incorporating *F. nucleatum* abundance, the ETBF signature, microbial metabolite profiles, tight-junction damage, circulating microbial products, IL-6/PGE2 activity, myeloid signatures, CAF/TGF-beta programs, hypoxia markers, and spatial T-cell exclusion may define resistance ecotypes more effectively than PD-L1 alone. Second, timing is crucial. Interventions that reduce harmful microbial translocation or dampen pathogenic inflammation may need to be implemented before or alongside checkpoint blockade, and broad-spectrum antibiotics, which risk depleting beneficial microbes, should not be used as crude immune adjuvants.

Accordingly, the clinical use of microbiome biomarkers should differ by MSI status: in MSI-H/dMMR CRC, they may refine prediction of primary or acquired resistance among patients already eligible for checkpoint blockade; in MSS CRC, they should be combined with evidence of T-cell priming and spatial niche disruption to identify tumors in which microbial or myeloid intervention could create, rather than merely preserve, checkpoint sensitivity.

Therefore, clinical trial design should shift towards resistance niche enrichment. Tumors rich in myeloid cells and with high IL-6/PGE2 activity are better suited to a combination of anti-inflammatory or myeloid repolarization strategies than T-cell-activating strategies alone. Tumors with stromal exclusion may require vascular or CAF-directed remodeling before checkpoint blockade can elicit meaningful T-cell activity. Microbiome-associated tumors should be evaluated through strain-level microbiome testing, metabolomics, and careful documentation of antibiotics, diet, bowel preparation, and prior treatments. These details, seemingly operational, are mechanistically critical because each can alter the microbiome and inflammatory baselines used to assess immunotherapy efficacy. The variability between REGONIVO and subsequent studies underscores the need to control these baseline factors when assessing immunotherapy efficacy.

Third, myeloid and stromal targets should be tested in biomarker-enriched populations. TAM-directed strategies, MDSC inhibition, macrophage repolarization, STING activation, COX-2/PGE2 modulation, IL-6 pathway inhibition, anti-angiogenic therapy, and TGF-beta or CAF-related strategies are all valid, but each carries inherent toxicity and context dependence. CRC-specific evidence has begun to support this direction: Li et al. showed that targeting MS4A4A on TAMs can restore CD8-positive T-cell-mediated antitumor immunity; Deng et al. reported that a STING agonist prodrug can reprogram TAMs and enhance CRC immunotherapy in preclinical models (47, 48). **Table 1** summarizes the main components of the proposed axis and its translational significance.

**Table 1:** Components of the microbiota-barrier-myeloid axis in CRC.

Component	Representative features	Immune effect	Candidate biomarkers	Translational considerations
Microbiota dysbiosis	*F. nucleatum*, ETBF, *Peptostreptococcus anaerobius*; altered SCFAs and bile acids	Epithelial stress, immune evasion, genotoxic or inflammatory signaling, and variable ICI effects	Microbial profiling, strain-level assays, and metabolomics	Avoid one-size-fits-all microbiome intervention; context and timing matter
Barrier disruption	Loss of ZO-1, claudins, and occludin; increased permeability; PAMP/DAMP leakage	Pattern-recognition receptor activation and chronic mucosal inflammation	Tight-junction and permeability markers; microbial products in tissue or blood	Barrier repair may need to accompany immunotherapy rather than replace it
Inflammatory cytokines	IL-6/STAT3, NF-kB, TNF-alpha, COX-2/PGE2, and IL-1R signaling	Epithelial survival, angiogenesis, myeloid recruitment, and suppressed T-cell priming	Cytokine panels, PGE2 signatures, phospho-STAT3, and spatial inflammation maps	Targeted anti-inflammatory strategies require toxicity-aware patient selection
Myeloid cells	TAMs, MDSCs, neutrophils, immature myeloid cells, and dysfunctional dendritic cells	Arginase, ROS, NO, IL-10, TGF-beta, VEGF, checkpoint ligands, and poor costimulation	Single-cell or spatial myeloid signatures; TAM or MDSC enrichment	Match repolarization or depletion strategies to the dominant myeloid state
Suppressive niches	Tregs, CAFs, CXCL12 barriers, TGF-beta-rich stroma, hypoxia/HIF-1alpha, and lactate	T-cell exclusion, metabolic stress, ECM remodeling, and reduced cytotoxicity	CAF/TGF-beta signatures, hypoxia markers, and spatial CD8 localization	Consider stromal and vascular barriers when sequencing combinations before ICI escalation

## 8 Conclusions and future perspectives

Immunotherapy resistance in colorectal cancer (CRC) is best viewed as an ecological problem. In MSS CRC, checkpoint blockade failure typically reflects the combined effects of dysbiosis, weakened mucosal barrier, chronic inflammatory circuits, suppressive myeloid cell populations, stromal exclusion, and metabolic stress. These mechanisms are interconnected: dysbiosis can damage the barrier; barrier damage allows translocation; microbial products amplify IL-6/STAT3, NF-κB, TNF-alpha, and COX-2/PGE2 signaling; these signals, in turn, recruit and educate myeloid cells, thereby limiting T-cell immunity.

Future research should move beyond simple tumor-fecal associations to paired microbiomic, metabolomic, spatial, single-cell, and clinical response datasets. The most useful biomarkers should be informative both spatially and functionally, revealing the location of microbes, barrier damage, inflammatory signaling, myeloid cells, fibroblasts, and T cells relative to the malignant epithelium. The therapeutic goal is not to sterilize the tumor or suppress all inflammation, but to reshape the dominant resistance niche, enabling antigen presentation, T-cell migration, and cytotoxic function without unacceptable toxicity. Rational immunotherapy for CRC requires mechanism-matched combination regimens, longitudinal monitoring, and adaptive trials, and should treat the microbiota-barrier-myeloid axis as a dynamic therapeutic target rather than a descriptive context.

## References

[1] Sung H Ferlay J Siegel RL Laversanne M Soerjomataram I Jemal A . Global Cancer Statistics 2020: GLOBOCAN estimates of incidence and mortality worldwide for 36 cancers in 185 countries. CA Cancer J Clin. (2021) 71:209–249. doi: 10.3322/caac.21660 33538338

[2] Dekker E Tanis PJ Vleugels JLA Kasi PM Wallace MB . Colorectal cancer. Lancet. (2019) 394:1467–80. doi: 10.1016/S0140-6736(19)32319-0 31631858

[3] André T Shiu KK Kim TW Jensen BV Jensen LH Punt C . Pembrolizumab in microsatellite-instability-high advanced colorectal cancer. N Engl J Med. (2020) 383:2207–2218. doi: 10.1056/NEJMoa2017699 33264544

[4] Le DT Durham JN Smith KN Wang H Bartlett BR Aulakh LK . Mismatch repair deficiency predicts response of solid tumors to PD-1 blockade. Science. (2017) 357:409–413. doi: 10.1126/science.aan6733 PMC5576142 28596308

[5] Ganesh K Stadler ZK Cercek A Mendelsohn RB Shia J Segal NH . Immunotherapy in colorectal cancer: rationale, challenges and potential. Nat Rev Gastroenterol Hepatol. (2019) 16:361–375. doi: 10.1038/s41575-019-0126-x PMC7295073 30886395

[6] Guinney J Dienstmann R Wang X de Reyniès A Schlicker A Soneson C . The consensus molecular subtypes of colorectal cancer. Nat Med. (2015) 21:1350–1356. doi: 10.1038/nm.3967 PMC4636487 26457759

[7] Wirbel J Pyl PT Kartal E Zych K Kashani A Milanese A . Meta-analysis of fecal metagenomes reveals global microbial signatures that are specific for colorectal cancer. Nat Med. (2019) 25:679–689. doi: 10.1038/s41591-019-0406-6 PMC7984229 30936547

[8] Thomas AM Manghi P Asnicar F Pasolli E Armanini F Zolfo M . Metagenomic analysis of colorectal cancer datasets identifies cross-cohort microbial diagnostic signatures and a link with choline degradation. Nat Med. (2019) 25:667–678. doi: 10.1038/s41591-019-0405-7 PMC9533319 30936548

[9] Wong SH Yu J . Gut microbiota in colorectal cancer: mechanisms of action and clinical applications. Nat Rev Gastroenterol Hepatol. (2019) 16:690–704. doi: 10.1038/s41575-019-0209-8 31554963

[10] Castellarin M Warren RL Freeman JD Dreolini L Krzywinski M Strauss J . Fusobacterium nucleatum infection is prevalent in human colorectal carcinoma. Genome Res. (2012) 22:299–306. doi: 10.1101/gr.126516.111 PMC3266037 22009989

[11] Kostic AD Gevers D Pedamallu CS Michaud M Duke F Earl AM . Genomic analysis identifies association of Fusobacterium with colorectal carcinoma. Genome Res. (2012) 22:292–298. doi: 10.1101/gr.126573.111 PMC3266036 22009990

[12] Kostic AD Chun E Robertson L Glickman JN Gallini CA Michaud M . Fusobacterium nucleatum potentiates intestinal tumorigenesis and modulates the tumor-immune microenvironment. Cell Host Microbe. (2013) 14:207–215. doi: 10.1016/j.chom.2013.07.007 PMC3772512 23954159

[13] Rubinstein MR Wang X Liu W Hao Y Cai G Han YW . Fusobacterium nucleatum promotes colorectal carcinogenesis by modulating E-cadherin/beta-catenin signaling via its FadA adhesin. Cell Host Microbe. (2013) 14:195–206. doi: 10.1016/j.chom.2013.07.012 23954158 PMC3770529

[14] Gur C Ibrahim Y Isaacson B Yamin R Abed J Gamliel M . Binding of the Fap2 protein of Fusobacterium nucleatum to human inhibitory receptor TIGIT protects tumors from immune cell attack. Immunity. (2015) 42:344–355. doi: 10.1016/j.immuni.2015.01.010 PMC4361732 25680274

[15] Yu T Guo F Yu Y Sun T Ma D Han J . Fusobacterium nucleatum promotes chemoresistance to colorectal cancer by modulating autophagy. Cell. (2017) 170:548–563:e16. doi: 10.1016/j.cell.2017.07.008 PMC5767127 28753429

[16] Bullman S Pedamallu CS Sicinska E Clancy TE Zhang X Cai D . Analysis of Fusobacterium persistence and antibiotic response in colorectal cancer. Science. (2017) 358:1443–1448. doi: 10.1126/science.aal5240 PMC5823247 29170280

[17] Zepeda-Rivera M Minot SS Bouzek H Wu H Blanco-Míguez A Manghi P . A distinct Fusobacterium nucleatum clade dominates the colorectal cancer niche. Nature. (2024) 628:424–432. doi: 10.1038/s41586-024-07182-w PMC11006615 38509359

[18] Jiang SS Xie YL Xiao XY Kang ZR Lin XL Zhang L . Fusobacterium nucleatum-derived succinic acid induces tumor resistance to immunotherapy in colorectal cancer. Cell Host Microbe. (2023) 31:781–797:e9. doi: 10.1016/j.chom.2023.04.010 37130518

[19] Wang X Fang Y Liang W Wong CC Qin H Gao Y . Fusobacterium nucleatum facilitates anti-PD-1 therapy in microsatellite stable colorectal cancer. Cancer Cell. (2024) 42:1729–1746:e8. doi: 10.1016/j.ccell.2024.08.019 39303724

[20] Wu S Rhee KJ Albesiano E Rabizadeh S Wu X Yen HR . A human colonic commensal promotes colon tumorigenesis via activation of T helper type 17 T cell responses. Nat Med. (2009) 15:1016–1022. doi: 10.1038/nm.2015 PMC3034219 19701202

[21] Dejea CM Fathi P Craig JM Boleij A Taddese R Geis AL . Patients with familial adenomatous polyposis harbor colonic biofilms containing tumorigenic bacteria. Science. (2018) 359:592–597. doi: 10.1126/science.aah3648 PMC5881113 29420293

[22] Chung L Thiele Orberg E Geis AL Chan JL Fu K DeStefano Shields CE . Bacteroides fragilis toxin coordinates a pro-carcinogenic inflammatory cascade via targeting of colonic epithelial cells. Cell Host Microbe. (2018) 23:203–214:e5. doi: 10.1016/j.chom.2018.01.007 PMC5954996 29398651

[23] Cao Y Wang Z Yan Y Ji L He J Xuan B . Enterotoxigenic Bacteroides fragilis promotes intestinal inflammation and malignancy by inhibiting exosome-packaged miR-149-3p. Gastroenterology. (2021) 161:1552–1566:e12. doi: 10.1053/j.gastro.2021.08.003 34371001

[24] O'Keefe SJD Li JV Lahti L Ou J Carbonero F Mohammed K . Fat, fibre and cancer risk in African Americans and rural Africans. Nat Commun. (2015) 6:6342. doi: 10.1038/ncomms7342 25919227 PMC4415091

[25] Yang J Wei H Zhou Y Szeto CH Li C Lin Y . High-fat diet promotes colorectal tumorigenesis through modulating gut microbiota and metabolites. Gastroenterology. (2022) 162:135–149:e2. doi: 10.1053/j.gastro.2021.08.041 34461052

[26] Neurath MF Artis D Becker C . The intestinal barrier: a pivotal role in health, inflammation, and cancer. Lancet Gastroenterol Hepatol. (2025) 10:573–92. doi: 10.1016/S2468-1253(24)00390-X 40086468

[27] Cheng S Han Z Dai D Li F Zhang X Lu M . Multi-omics of the gut microbial ecosystem in patients with microsatellite-instability-high gastrointestinal cancer resistant to immunotherapy. Cell Rep Med. (2024) 5:101355. doi: 10.1016/j.xcrm.2023.101355 38194971 PMC10829783

[28] Liu Y Wong CC Ding Y Gao M Wen J Lau HCH . Peptostreptococcus anaerobius mediates anti-PD1 therapy resistance and exacerbates colorectal cancer via myeloid-derived suppressor cells in mice. Nat Microbiol. (2024) 9:1467–1482. doi: 10.1038/s41564-024-01695-w PMC11153135 38750176

[29] Gao Y Bi D Xie R Li M Guo J Liu H . Fusobacterium nucleatum enhances the efficacy of PD-L1 blockade in colorectal cancer. Signal Transduct Target Ther. (2021) 6:398. doi: 10.1038/s41392-021-00795-x 34795206 PMC8602417

[30] Grivennikov S Karin E Terzic J Mucida D Yu GY Vallabhapurapu S . IL-6 and Stat3 are required for survival of intestinal epithelial cells and development of colitis-associated cancer. Cancer Cell. (2009) 15:103–113. doi: 10.1016/j.ccr.2009.01.001 PMC2667107 19185845

[31] Greten FR Eckmann L Greten TF Park JM Li ZW Egan LJ . IKKbeta links inflammation and tumorigenesis in a mouse model of colitis-associated cancer. Cell. (2004) 118:285–296. doi: 10.1016/j.cell.2004.07.013 15294155

[32] Wang D DuBois RN . Eicosanoids and cancer. Nat Rev Cancer. (2010) 10:181–93. doi: 10.1038/nrc2809 20168319 PMC2898136

[33] Xie M Yuan K Zhang Y Zhang Y Zhang R Gao J . Tumor-resident probiotic Clostridium butyricum improves aPD-1 efficacy in colorectal cancer models by inhibiting IL-6-mediated immunosuppression. Cancer Cell. (2025) 43:1885–1901:e10. doi: 10.1016/j.ccell.2025.07.012 40780216

[34] van Baarle L De Simone V Schneider L Santhosh S Abdurahiman S Biscu F . IL-1R signaling drives enteric glia-macrophage interactions in colorectal cancer. Nat Commun. (2024) 15:6079. doi: 10.1038/s41467-024-50438-2 39030280 PMC11271635

[35] Zhang L Li Z Skrzypczynska KM Fang Q Zhang W O'Brien SA . Single-cell analyses inform mechanisms of myeloid-targeted therapies in colon cancer. Cell. (2020) 181:442–459:e29. doi: 10.1016/j.cell.2020.03.048 32302573

[36] Veglia F Sanseviero E Gabrilovich DI . Myeloid-derived suppressor cells in the era of increasing myeloid cell diversity. Nat Rev Immunol. (2021) 21:485–98. doi: 10.1038/s41577-020-00490-y 33526920 PMC7849958

[37] Jaillon S Ponzetta A Di Mitri D Santoni A Bonecchi R Mantovani A . Neutrophil diversity and plasticity in tumour progression and therapy. Nat Rev Cancer. (2020) 20:485–503. doi: 10.1038/s41568-020-0281-y 32694624

[38] Pelka K Hofree M Chen JH Sarkizova S Pirl JD Jorgji V . Spatially organized multicellular immune hubs in human colorectal cancer. Cell. (2021) 184:4734–4752:e20. doi: 10.1016/j.cell.2021.08.003 PMC8772395 34450029

[39] Schürch CM Bhate SS Barlow GL Phillips DJ Noti L Zlobec I . Coordinated cellular neighborhoods orchestrate antitumoral immunity at the colorectal cancer invasive front. Cell. (2020) 182:1341–1359:e19. doi: 10.1016/j.cell.2020.07.005 PMC7479520 32763154

[40] Qi J Sun H Zhang Y Wang Z Xun Z Li Z . Single-cell and spatial analysis reveal interaction of FAP+ fibroblasts and SPP1+ macrophages in colorectal cancer. Nat Commun. (2022) 13:1742. doi: 10.1038/s41467-022-29366-6 35365629 PMC8976074

[41] Tauriello DVF Palomo-Ponce S Stork D Berenguer-Llergo A Badia-Ramentol J Iglesias M . TGFbeta drives immune evasion in genetically reconstituted colon cancer metastasis. Nature. (2018) 554:538–543. doi: 10.1038/nature25492 29443964

[42] Calon A Espinet E Palomo-Ponce S Tauriello DVF Iglesias M Céspedes MV . Dependency of colorectal cancer on a TGF-beta-driven program in stromal cells for metastasis initiation. Cancer Cell. (2012) 22:571–584. doi: 10.1016/j.ccr.2012.08.013 PMC3512565 23153532

[43] Valdeolivas A Amberg B Giroud N Richardson M Gálvez EJC Badillo S . Profiling the heterogeneity of colorectal cancer consensus molecular subtypes using spatial transcriptomics. NPJ Precis Oncol. (2024) 8:10. doi: 10.1038/s41698-023-00488-4 38200223 PMC10781769

[44] Overman MJ McDermott R Leach JL Lonardi S Lenz HJ Morse MA . Nivolumab in patients with metastatic DNA mismatch repair-deficient or microsatellite instability-high colorectal cancer (CheckMate 142): an open-label, multicentre, phase 2 study. Lancet Oncol. (2017) 18:1182–1191. doi: 10.1016/S1470-2045(17)30422-9 PMC6207072 28734759

[45] Fukuoka S Hara H Takahashi N Kojima T Kawazoe A Asayama M . Regorafenib plus nivolumab in patients with advanced gastric or colorectal cancer: an open-label, dose-escalation, and dose-expansion phase Ib trial (REGONIVO, EPOC1603). J Clin Oncol. (2020) 38:2053–2061. doi: 10.1200/JCO.19.03296 32343640

[46] Eng C Kim TW Bendell J Argilés G Tebbutt NC Di Bartolomeo M . Atezolizumab with or without cobimetinib versus regorafenib in previously treated metastatic colorectal cancer (IMblaze370): a multicentre, open-label, phase 3, randomised, controlled trial. Lancet Oncol. (2019) 20:849–861. doi: 10.1016/S1470-2045(19)30027-0 31003911

[47] Li Y Shen Z Chai Z Zhan Y Zhang Y Liu Z . Targeting MS4A4A on tumour-associated macrophages restores CD8+ T-cell-mediated antitumour immunity. Gut. (2023) 72:2307–2320. doi: 10.1136/gutjnl-2022-329147 PMC10715532 37507218

[48] Deng A Fan R Hai Y Zhuang J Zhang B Lu X . A STING agonist prodrug reprograms tumor-associated macrophage to boost colorectal cancer immunotherapy. Theranostics. (2025) 15:277–299. doi: 10.7150/thno.101001 PMC11667223 39744236

